# Trust-Aware and Fuzzy Logic-Based Reliable Layering Routing Protocol for Underwater Acoustic Networks

**DOI:** 10.3390/s23239323

**Published:** 2023-11-22

**Authors:** Duoliang Han, Xiujuan Du, Lijuan Wang, Xiuxiu Liu, Xiaojing Tian

**Affiliations:** 1 Department of Computer, Qinghai Normal University, Xining 810008, China; 202033341016@stu.qhnu.edu.cn (D.H.); 20231075@qhnu.edu.cn (L.W.); lxx@qhnu.edu.cn (X.L.); 202133341004@stu.qhnu.edu.cn (X.T.); 2 Key Laboratory of the Internet of Things of Qinghai Province, Xining 810008, China; 3 The State Key Laboratory of Tibetan Intelligent Information Processing and Application, Xining 810008, China

**Keywords:** underwater acoustic networks, trust-aware, fuzzy logic, reliable routing

## Abstract

Routing protocols based on trust mechanisms have been widely investigated for wireless sensor networks, and the works have achieved good results, while there are few works on trusted routing for underwater acoustic networks (UANs). However, trust-aware routing is the key to improving the packet delivery rate and the energy efficiency of UANs. Therefore, inspired by the theory of trust evaluation, a trust-aware and fuzzy logic-based reliable layering routing protocol (TAFLRLR) is proposed. In the TAFLRLR protocol, to avoid the problem of the void area and improve the transmission reliability, the candidate nodes of the next-hop forwarding nodes are determined according to the layers of neighbor nodes. Moreover, a fuzzy logic-based trust evaluation mechanism (FLTEM) is provided, which employs the fuzzy comprehensive evaluation decision model to calculate the comprehensive trust value for underwater sensor nodes. Further, the node density of a candidate node and its comprehensive trust value are taken as the input of a fuzzy control system and the forwarding probability (FP) of the node is taken as the output, and the candidate node with the highest FP is selected as the best forwarding node. Simulation results illustrate the superiority and effectiveness of the TAFLRLR protocol in terms of energy efficiency, routing reliability, and transmission reliability.

## 1. Introduction

In recent years, underwater acoustic networks (UANs) have been widely used in marine pollution monitoring, aided navigation, resource exploration, and military fields [1]. UANs are deployed underwater and composed of numerous sensor nodes, autonomous underwater vehicles, and unmanned underwater vehicles [2,3]. UANs use sensors to collect the physical information of the water, such as temperature, depth, and salinity, and transmit the information to a sink node in a multi-hop manner. Then, the sink node sends the information to the server using the radio signal for data analysis.

Usually, research concerning UANs includes topology control, node localization, routing and MAC protocol, reliable transmission, etc. However, compared with terrestrial wireless sensor networks, the following characteristics of UANs bring significant challenges for node deployment, protocol design, and reliable transmission.

(1) The propagation speed of the acoustic waves in water is 1500 m/s, which leads to a long propagation delay.
(2) The channel error rate is high due to the influence of the Doppler effect, path loss, ship noises, etc.
(3) The acoustic signals can decay with increasing transmission distance when transmitted underwater; the exponential decay factor increases with increasing frequency; thus, low-frequency signals are more preferable for long-range communications, which results in a low bit rate.
(4) The underwater node is powered by the battery, and it is difficult to recharge or replace the battery in harsh underwater environments, which results in the energy of the node being limited. However, underwater acoustic modems consume more energy in packet transmission than terrestrial radio frequency modems.
(5) The nodes move autonomously or with the water flow or other activities, which leads to a dynamically changing network topology [4,5,6].

These characteristics have a significant effect on the protocol design as well as the performance of the designed protocol. In addition, the opening and sharing of underwater acoustic channels also bring about great challenges to the reliable transmission of UANs. Nodes of UANs are deployed underwater and are unguarded, and they are easily captured as malicious nodes (MNs), which expose the network to threats and attacks. For example, an MN may flood HELLO packets (HELLO packets are also called beacon messages, which are usually used for neighbor discovery and routing maintenance. In our proposed protocol, HELLO packets are also used for configuring the layer of nodes. The details are provided in Section 3) in the network. The malicious HELLO packets have the following negative impacts. (1) Many collisions occur in the network, which decreases the packet delivery rate. (2) Other nodes are subject to forward malicious HELLO packets, which results in low energy efficiency. (3) Data packets from sensor nodes are transmitted probably to the MNs instead of the sink node. The above-mentioned is a typical HELLO packet flooding attack. Beyond that, there are black hole attacks, selective forwarding attacks, etc. All of these can lead to severe network bandwidth consumption, energy consumption, and channel utilization reduction. Therefore, it is necessary to design trust-aware routing protocols to defend against MN attacks and threats to UANs. In this view, some defensive mechanisms and secure routing protocols, such as an energy-efficient key agreement mechanism [7], a secure and reliable multi-path transmission scheme [8], a secure routing scheme [9], and a secure routing protocol based on depth [10] have been investigated and developed. The existing defensive and security mechanisms can be divided roughly into intrusion protection, intrusion detection, and intrusion tolerance mechanisms. In intrusion tolerance mechanisms, trust evaluation is not only a powerful means to ensure the reliability of routing but also an effective supplement to encryption algorithms and authentication mechanisms in the view of security. Jiang et al. studied the problem of trust between nodes in UANs, analyzed the existing trust management mechanism, and proposed a trust cloud model according to the characteristics of UANs [11]. The trust cloud model can improve the calculation accuracy of trust between nodes and the communication success rate. In [12], Krishnaswamy et al. proposed a scheme to select the trusted cluster heads and their member nodes based on fuzzy logic. The proposed scheme uses a lightweight XOR encryption algorithm to authenticate the nodes in the cluster and calculates the trust value based on the residual energy of the node, the distance, and relative mobility between nodes, and then uses fuzzy logic to select trusted nodes based on the trust value. In both [11,12], trust management is used to improve the reliability of transmission.

Inspired by [11,12], in this paper, we propose a fuzzy logic-based trust evaluation mechanism (FLTEM) for underwater sensor nodes. FLTEM uses the fuzzy comprehensive evaluation decision model to calculate the comprehensive trust value of nodes to identify MNs and improve the reliability of the network. Moreover, we designed a trust-aware and fuzzy logic-based reliable layering routing (TAFLRLR) protocol using FLTEM. We employ a fuzzy control system (FCS) to calculate the forwarding probability (FP) and select the best next-hop node.

The main contributions of this paper can be summarized as follows:

(1) A layering algorithm is proposed to configure a layer for each node according to the minimum hop count, which improves the delivery rate of packets by avoiding the problem of void area.
(2) A fuzzy logic-based trust evaluation mechanism for underwater nodes, called FLTEM, is proposed. Considering the residual energy and different forwarding behaviors between HELLO packets and DATA packets, a fuzzy comprehensive evaluation decision model is introduced to calculate the direct trust value (DTV) of a neighbor node, and then the indirect trust value (ITV) is calculated according to the DTVs of the public neighbor nodes. Finally, the comprehensive trust value (CTV) can be obtained based on the DTV and ITV, and CTV can be used to effectively identify the MNs in the network. For instance, node n calculates the *DTV_n,a_* for its neighboring node a based on the communication behavior of node a, node a calculates the *DTV_a,k_* for its neighboring node k based on the communication behavior of node k; thus, the ITV of node k for node n is *DTV_n,a_ × DTV_a,k_*. The CTV is the weighted sum of DTV and ITV. The calculation of DTV, ITV, and CTV is detailed in Section 4.
(3) A trust-aware and fuzzy logic-based reliable layering routing protocol, called TAFLRLR, is proposed to achieve transmission reliability. TAFLRLR takes the CTV and node density (ND) of a candidate node as the input of FCS and the FP as the output. The candidate node with the maximum FP will be selected as the best next-hop node.
(4) Simulation results in NS3 show that the TAFLRLR protocol has superior performance in terms of transmission reliability, routing reliability and energy efficiency.

The remainder of this paper is organized as follows. Section 2 presents several common threats and attacks faced by UANs. The network model and network initialization are introduced in Section 3. The proposed FLTEM and TAFLRLR are described in detail in Section 4 and Section 5, respectively. In Section 6, we evaluate the performance of the TAFLRLR protocol via NS3 simulations. In Section 7, the paper is summarized, and future research is proposed.

## 2. Attacks in Underwater Acoustic Networks

As mentioned previously, the opening and sharing of the underwater acoustic channel make UANs vulnerable to various threats and attacks from MNs. Three common threats and attacks in UANs are as follows [13,14,15,16].

(1) A HELLO packet flooding attack occurs when an MN sends HELLO packets with higher power than that from a good node, which causes the nodes receiving the HELLO packet to consider the MN as their neighbor. In the subsequent data forwarding, the nodes that have received the HELLO packets from the MN may choose the MN as the best next-hop node and send the data packets to the MN. However, the transmission power of the normal nodes is less than that of the MN, which means that the data packets are unable to reach either the sink node or the MN.
(2) Another HELLO packet flooding attack is considered in this paper. It occurs when an MN frequently floods HELLO packets in the network, which interferes heavily with the normal communication in UANs, intensifies the packet re-transmission, increases the network energy consumption, and reduces the packet delivery rate.
(3) In a selective forwarding attack, the MN masquerades as a normal node in the network and drops packets with a certain probability, resulting in packet loss. So, selective forwarding attacks exacerbate packet re-transmissions, reduce the packet delivery rate, and consume much energy, which is unfavorable for energy-limited UANs.

The above attacks severely waste the resources of the network, such as bandwidth, energy, and channel utilization. Therefore, a fuzzy comprehensive evaluation decision model is introduced to establish a trust evaluation mechanism for underwater nodes and identify the MNs in the network to mitigate the threats caused by the above attacks and ensure reliable routing.

## 3. Network Model and Network Initialization

### 3.1. Network Model

**Figure 1:**
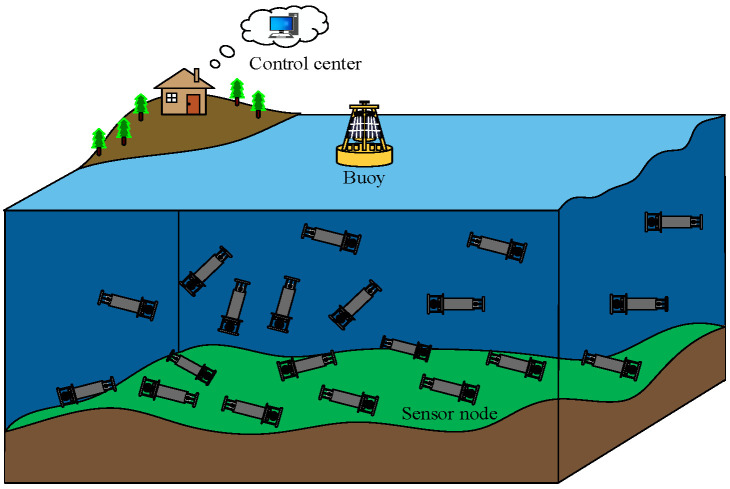
Network model.

The considered 3D network model is shown in Figure 1, where one source node is deployed underwater and it is responsible for periodically collecting and sending data. Numerous nodes (forwarding nodes) are randomly deployed underwater to receive a DATA packet and transmit it from the source node [17]. A sink node deployed on the water surface collects DATA packets and delivers the packets to the server in a data center. For understanding, we made the following assumptions.

(1) Each node in the network has a unique ID.
(2) All the legal nodes in the network are isomorphic and have the same parameter settings, such as receiving power, transmitting power, idle power, sleeping power, and communication radius.
(3) The source node generates packets periodically.
(4) The CTV of each node in the network is initialized to 0.5 in the network initialization. Moreover, the value range of the CTV/DTV/ITV is [0, 1].

### 3.2. Network Initialization

In the initialization phase of the network, the sink node starts to broadcast periodically HELLO packets. A HELLO packet is identified by using the sequence number (HELLO_seq_num) and involves some key information of the sending node, such as ID, layer, and residual energy. The information of the sending node is updated hop-by-hop, and the node receiving the HELLO packet updates its layer and neighbor information table according to the information from the received packets, which is described later. The structure of a HELLO packet is shown in Table 1 [17], in which SN represents the sending node and RN represents the receiving node. S_ID and R_ID are the IDs of the sending node and receiving node, respectively.

When an underwater node hears a HELLO packet, it stores the ID, layer, and residual energy of the sending node in the neighbor information table and records the HELLO_seq_num. The receiving node compares the layer of the sending node with its layer to determine whether to update its layer and forward the HELLO packet. Only the nodes with larger layers than the sending node are allowed to update and forward the HELLO packet, which avoids packet collisions and saves energy. Significantly, the information in the HELLO packet is updated hop-by-hop. In this way, the nodes in the network can discover their neighbor nodes. The sink node periodically floods the HELLO packets, which allow the underwater nodes to update their neighbor information tables in time. Each node can only forward the HELLO packet once within a flooding cycle period. In this paper, the flooding cycle period *T_flood_* of the HELLO packets is defined according to the communication radius and the average movement speed of underwater nodes:

(1) $$T_{flood}=\frac{\frac{R}{2}}{V_{ave\_mov}}+Rand()$$

where *R* is the communication radius of the node, *V_ave_mov_* denotes the average moving speed of nodes, and *Rand* () is the random function with a range of (0, 10].

As mentioned earlier, each node maintains a neighbor information table in which the ID, the layer *L_n_*, the residual energy *E_r_*, and RH_flag of the neighbors are recorded. The structure of the neighbor information table is shown in Table 2, in which the RH_Flag is used to indicate whether HELLO packets have been received from neighboring nodes and has been described in detail in Section 4. *N_n_c_* denotes the number of HELLO packets forwarded normally, *N_a_c_* denotes the number of HELLO packets forwarded abnormally, *N_n_d_* represents the number of DATA packets forwarded normally, and *N_a_d_* represents the number of the DATA packets forwarded abnormally. The definitions of HELLO/DATA packets normally/abnormally forwarded have been given in Section 4.

All the information stored in the neighbor information table will be used to calculate DTV and select the best forwarding node.

### 3.3. Layering Algorithm

To avoid the void area routing problem and improve transmission reliability, a layer update algorithm is proposed, which is shown in Algorithm 1.

In the TAFLRLR protocol, each node maintains a layer that represents the hop count from the node to the sink. The layer (*L_sink_*) of the sink node is set to 0, and the layer (*L_cur_*) of other nodes is initialized to *0xFF* [17,18,19]. The layer is the only basis for determining whether a node is a candidate forwarding node. When a node receives a HELLO packet, it updates its layer according to the layer (*L_send_*) of the sending node in the header of the packet. As shown in Algorithm 1, a layer aging timer (i.e., layer_aging_timer) is set for each node. The layer aging timer is initialized to the flooding period *T_flood_*. Received_HELLO_seq_num represents the sequence number of the HELLO packet received by the node.

After the layers of all the nodes are configured, the HELLO packets are transmitted only along the paths from nodes with lower layers to nodes with higher layers. On the contrary, DATA packets are transmitted along the paths from nodes with higher layers to nodes with lower layers until the sink node.
   **Algorithm 1:** Layer update procedure1:Network initialization;2:*L_sink_* = 0;3:*L_cur_* = 0xFF;4:HELLO_seq_num = 0;5:When node N within the communication range of node M receives a HELLO packet;6:**if** (Received_HELLO_seq_num != HELLO_seq_num)7:  **if** (*L_cur_* == 255) **then**8:    *L_cur_* = *L_send_* + 1;9:    Node N updates the header and load information of the HELLO packet and forwards it;10:  **else**
11:    **If** (*layer_aging_timer* > 0 and *L_cur_* < *L_send_* +1) **then**12:       Node N discards the HELLO packet and does not update its layer;13:    **else**14:       *L_cur_* = *L_send_* + 1;15:       Node N updates header and load information of the HELLO packet and forwards it;16:    **end if**17:  **end if**
18:**else**19:  Node N updates its neighbor information table;20:**end if**

## 4. Trust Evaluation Mechanism

Trust evaluation is used to evaluate the reliability of nodes in networks based on their behavior and has been widely investigated in networks such as WSNs [20,21]. In WSNs, the behavior of sensor nodes includes HELLO packet forwarding, DATA packet forwarding, etc. Evaluating the behavior of nodes to identify MNs in the network provides a strong guarantee for the secure and reliable communication of WSNs.

**Figure 2:**
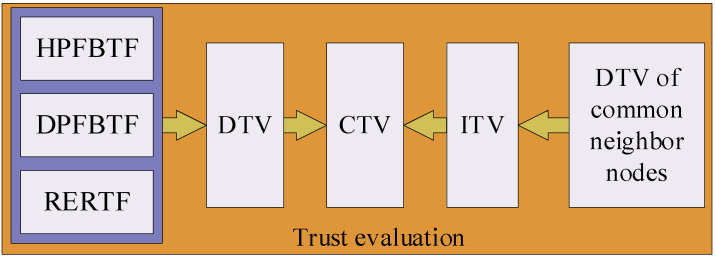
The framework of FLTEM.

Compared with nodes in WSNs, underwater nodes are more likely to be captured, which makes UAN communication vulnerable to MN attacks. Therefore, it is essential to establish a trust evaluation mechanism for the nodes to identify MNs and ensure the reliable communication of the UANs. In this paper, a fuzzy logic-based trust evaluation mechanism (FLTEM) is presented. In FLTEM, nodes are evaluated according to their residual energy and forwarding behavior. The DTV of the nodes is calculated by using a fuzzy comprehensive evaluation decision model, and the average of the DTVs from multiple common neighbor nodes is taken as the ITV of the node [22]. Furthermore, the reliability of a node is denoted by the CTV, which is determined according to the DTV and ITV. The framework of FLTEM is shown in Figure 2, where HPFBTF represents the trust factor of HELLO packet forwarding behavior, DPFBTF is the trust factor of DATA packet forwarding behavior, and RERTF is the trust factor of residual energy. DTV, ITV, and CTV will be described in detail later in this section.

### 4.1. Direct Trust Value of Nodes

Based on the membership theory of fuzzy mathematics, the fuzzy comprehensive evaluation decision converts the qualitative evaluation of the evaluation object into a quantitative evaluation and performs a comprehensive evaluation with multiple constraints on the evaluation object. In addition, the fuzzy comprehensive evaluation decision model has the characteristics of accurate results and strong systematization, which are suitable for solving uncertain problems, so the DTVs of nodes are calculated based on the fuzzy comprehensive evaluation decision model. The steps to calculate the DTV are given as follows.

(1) Construct the factor set $U=\{u_{1},u_{2},u_{3}\}$, where *u*_1_, *u*_2_, and *u*_3_ represent HPFBTF, DPFBTF, and RERTF, respectively.

HPFBTF is calculated according to the number of HELLO packets normally and abnormally forwarded by a node. For understanding, we provide the following definition.

**Definition** **1.** *The behavior of abnormally forwarding HELLO packets means that the node does not forward HELLO packets according to the rules.*

In Beta reputation systems designed for sensor networks, Ganeriwal et al. fitted the reputation distribution and Beta distribution using the Bayesian formula and concluded that the reputation of sensor nodes obeys the Beta distribution [16,23]. Therefore, the trust factor of the nodes can be obtained by calculating the expectation of Beta distribution [24]. Assuming that the trust factor distribution of HELLO packet forwarding behavior of node k is denoted by *h*, then according to the expectation *E* of Beta distribution, the *HPFBTF_n,k_* of node k calculated using node n can be expressed as

(2) $$HPFBTF_{n,k}=E(h)=\frac{N_{n\_c}+1}{N_{n\_c}+N_{a\_c}+2}$$

where *N_n_c_* and *N_a_c_* represent the number of HELLO packets forwarded normally and abnormally by node k, respectively.

As mentioned earlier, a RH_Flag field is set in the neighbor table for each neighboring node to indicate whether the node has received a HELLO packet forwarded by the neighbor node. At the beginning of each HELLO cycle, the node sets the RH_Flag field for each neighbor node to “0”. Specifically, when node R receives a HELLO packet from node S for the first time within the cycle, it sets the RH_Flag to “1”. Afterwards, when node R receives one more HELLO packet forwarded by node S within the same cycle, node R determines whether the HELLO packet has been received from node S by determining the RH_Flag in the neighbor information table. If RH_Flag = 1, we think node S forwards the HELLO packet abnormally once.; in this case, *N_a_c_* = *N_a_c_* + 1; otherwise, *N_n_c_ = N_n_c_* + 1.

DPFBTF is calculated by using the number of DATA packets forwarded normally and abnormally by a node. For understanding, we provide the following definition.

**Definition** **2.** *The behavior of abnormally forwarding DATA packets means that an MN is selected as a forwarding node; however, the MN discards the received packets instead of forwarding them, or the MN does not forward the received DATA packets according to the specific routing rules. For example, a packet is forwarded to a node with a higher layer than that of the forwarder.*

As mentioned earlier, the trust factor of a node can be obtained by calculating the expectation of the Beta distribution. Assuming that the trust factor distribution of the DATA packet forwarding behavior of node k is *d*, then according to the expectation *E* of Beta distribution, the *DPFBTF_n,k_* of node k calculated by node n can be expressed as

(3) $$DPFBTF_{n,k}=E(d)=\frac{N_{n\_d}+1}{N_{n\_d}+N_{a\_d}+2}$$

where *N_n_d_* and *N_a_d_* represent the number of DATA packets forwarded normally and abnormally by node k, respectively.

The detection of abnormal forwarding of data packets mainly includes two aspects. On the one hand, if a node in the network hears a data packet in which the layer of the receiver is greater than that of the sender, it implies that the sender may be an MN. In this case, *N_a_d_* = *N_a_d_* + 1. Otherwise, *N_n_d_* = *N_n_d_* + 1. On the other hand, after forwarding a data packet, the node switches to the listening mode to determine whether the selected next-hop node is an MN. If no data packet is heard from the next-hop node during RTT (Round-Trip Time), it implies that the selected next-hop node may be an MN. In this case, *N_a_d_* = *N_a_d_* + 1. Otherwise, *N_n_d_* = *N_n_d_* + 1.

RERTF: In UANs, the energy of nodes is limited. Unbalanced energy consumption has a great impact on network performance, such as the network lifetime. The reasonable and balanced use of node energy can effectively improve network performance. However, the energy consumption of the nodes is not taken into account in some of the existing trust mechanisms. Consequently, the residual energy of nodes is considered a trust factor (i.e., *RERTF*) to balance the energy consumption of UANs. Assuming that the initial energy of a node is *E_init_*, and the residual energy of node k is *E_resi_*, then the *RERTF*_n, k_ of node k calculated by node n can be expressed as

(4) $$REFTF_{n,k}=\frac{E_{resi}}{E_{init}}$$

**Figure 3:**
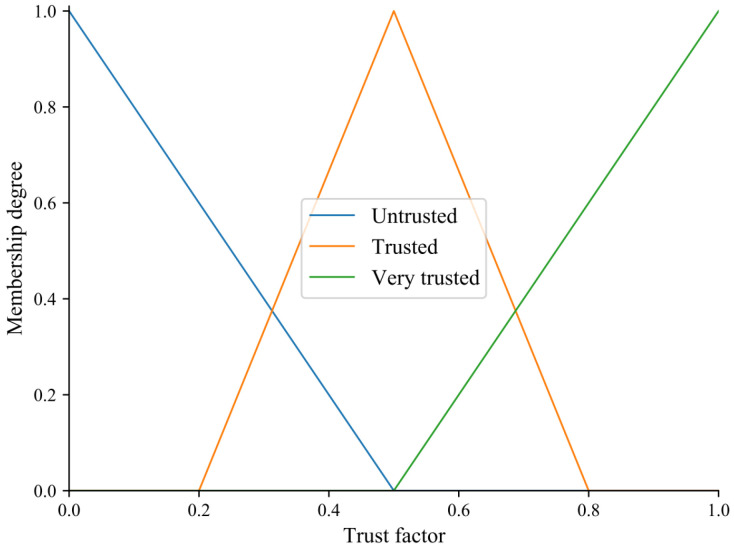
The membership function of the elements of the evaluation set.

(1) Construct the evaluation set $V=\{v_{1},v_{2},v_{3}\}$ to indicate the degree of trust for each trust factor (i.e., *u*_1_, *u*_2_, *u*_3_), where *v*_1_, *v*_2_, and *v*_3_ represent untrusted, trusted, and very trusted, respectively.
(2) Get the membership degree matrix *R*. Membership degree of trust factors *u*_1_, *u*_2_ and *u*_3_ is obtained via the triangle membership function, which is shown in Figure 3.

Membership degree matrix *R* is defined as

(5) $$R=\left\{\begin{array}{ccc} r_{11} & r_{12} & r_{13}\\ r_{21} & r_{22} & r_{23}\\ r_{31} & r_{32} & r_{33} \end{array}\right\}$$

In *R*, each element *r_ij_* is the membership degree of *u_i_* to the evaluation set element *v_j_*.

(1) Obtain the evaluation result. Given a fuzzy subset $V=(w_{1},w_{2},w_{3})$, $\sum_{i=1}^{3}w_{i}=1$, where *w_i_* represents the weight of the *i*th trust factor, and the weight is adjustable. Then, by utilizing the weighted average model M(⋅,+) [25], the evaluation result *B* can be obtained:
  (6) $$\begin{array}{c} B=W\circ R=(w_{1},w_{2},w_{3})\circ \left\{\begin{array}{ccc} r_{11} & r_{12} & r_{13}\\ r_{21} & r_{22} & r_{23}\\ r_{31} & r_{32} & r_{33} \end{array}\right\}\\ =(b_{1},b_{2},b_{3}) \end{array}$$

By normalizing *B*, we obtain the evaluation result $B^{'}$

(7) $$B^{'}=(\frac{b_{1}}{\delta },\frac{b_{2}}{\delta },\frac{b_{3}}{\delta })$$

where $\delta =\sum_{i=1}^{3}b_{i}$, $B^{'}$ is considered as a weight. Given

(8) $$V={\left(0.15,0.5,0.95\right)}^{Τ}$$

Then, the DTV of node k calculated by node n can be obtained as Equation (9)

(9) $$DTV_{n,k}=B^{'}V$$

### 4.2. Indirect Trust Value of Nodes

**Figure 4:**
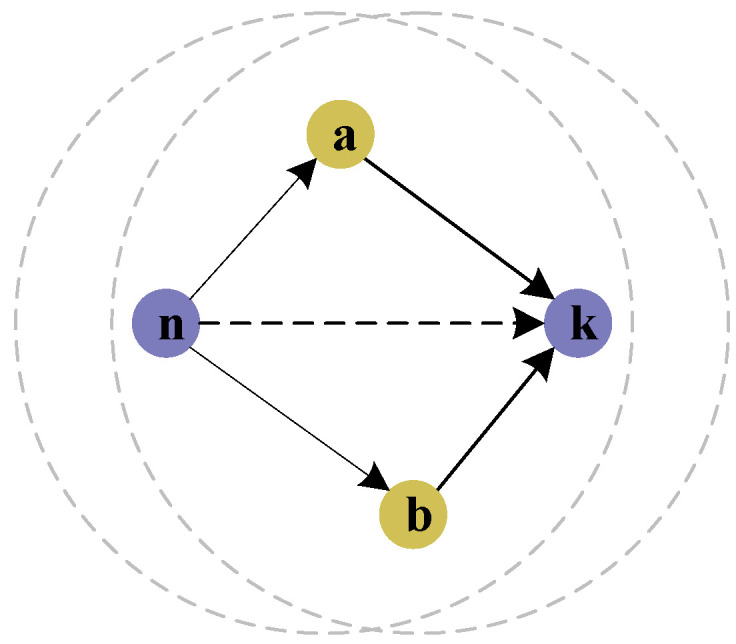
Schematic diagram of the ITV.

The ITV of the node is calculated according to the DTVs of their common neighbors. However, if the common neighbor node is an MN, the MN may tamper with the DTV of node k. Therefore, to prevent the network from being attacked, the average value of the DTVs of common neighbor nodes is considered the ITV of the evaluated node. Take Figure 4 as an example for further explanation. It can be seen that node a and node b are the common neighbor nodes of node n and node k.

Let the DTVs of node a and node b calculated by node n be *DTV_n,a_* and *DTV_n,b_*, respectively. Let the DTVs of node k calculated by node a and b be *DTV_a,k_* and *DTV_b,k_*, respectively. The ITV of node k calculated by node n can be expressed as

(10) $$ITV_{n,k}=\frac{DTV_{n,a}DTV_{a,k}+DTV_{n,b}DTV_{b,k}}{2}$$

Moreover, let the set $P=\{p_{1},p_{2},p_{3},\ldots ,p_{e}\}$ be the common neighbor nodes of node n and node k, where *e* is the number of common neighbor nodes, then the generalized form of the ITV of node k calculated by node n is

(11) $$ITV_{n,k}=\frac{1}{e}\sum_{m=1}^{e}\left(DTV_{n,p}\times DTV_{p,k}\right)$$

### 4.3. Comprehensive Trust Value of Nodes

To further improve the discrimination accuracy of MNs, the CTV is calculated according to the DTV and ITV of the nodes and can be expressed as

(12) $$CTV_{n,k}=\alpha DTV_{n,k}+\beta ITV_{n,k}$$

where *α* and *β* are weight coefficients, and α+β=1.

## 5. TAFLRLR Protocol

### 5.1. Overview of the TAFLRLR Protocol

**Figure 5:**
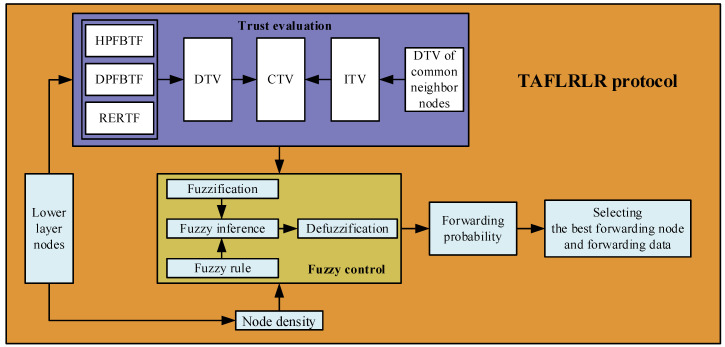
The overall framework of the TAFLRLR protocol.

Aiming at the characteristics and challenges of UANs mentioned in the previous section, a trust-aware and fuzzy logic-based reliable layering routing protocol is proposed to achieve routing reliability and energy efficiency. The framework of TAFLRLR is shown in Figure 5. The TAFLRLR protocol has the following features.

(1) With the TAFLRLR protocol, a layer is configured for each node, and DATA packets are transmitted layer by layer from a node with a higher layer to the sink node with layer 0, so the reliability of the routing is guaranteed.
(2) The TAFLRLR is essentially a single-path routing, in which one path is established between a source node and the sink node. In each hop, the forwarding node is determined by the sender of the previous hop. Compared with multi-path routing, TAFLRLR can decrease the probability of packet collision and effectively reduce energy consumption.
(3) The FLTEM mechanism is proposed for the trust evaluation of nodes. Specifically, with the consideration of HELLO packet forwarding behavior, DATA packet forwarding behavior, and residual energy, a fuzzy comprehensive evaluation decision model is introduced to calculate DTV for each node. In addition, combined with the DTV and ITV, the CTV is calculated to identify the MNs in UANs as well as select the best forwarding node.
(4) The best forwarding node in the TAFLRLR is decided by the forwarding probabilities (FPs) of candidate forwarding nodes; the candidate forwarding node with the maximum FP is chosen as the best forwarding node. To improve the performance of the routing for UANs, the FP is calculated through a fuzzy control system (FCS) since the FCS has many advantages, such as low computational complexity and excellent adaptability [17,26]. In the FCS, the NDs and the CTVs of candidate nodes are taken as the input variables of the FCS, and the outputs of the FCS are the FPs of the input nodes.

### 5.2. Best Forwarding Node Selection and Data Forwarding

In this subsection, the calculation of *ND* and *FP* is presented in detail.

In the TAFLRLR protocol, the sending node selects all the nodes whose layer is 1 smaller than its layer in the neighbor information table as candidate forwarding nodes and utilizes an FCS to calculate the FPs of candidate nodes according to the ND and CTV of the candidate nodes [17]. The calculation of the CTV is presented in detail in Section 4. The calculation of *ND* is given in Equation (13).

(13) $$ND=\frac{N_{L-1}}{N-2}\in (0,1)$$

where *N*_*L*−1_ denotes the number of neighbor nodes whose layer is 1 lower than the layer of the candidate forwarding node, and *N* is the number of all nodes in the network.

**Figure 6:**
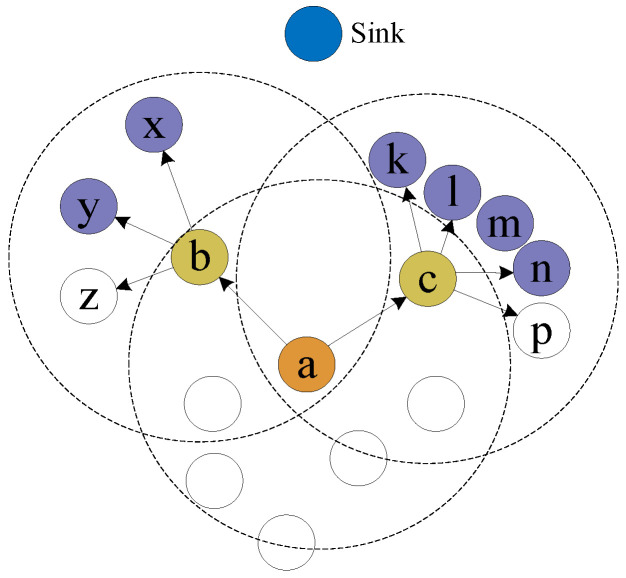
Network topology.

Next, an example is given to explain the calculation of *ND*, which is shown in Figure 6. In Figure 6, there are 17 nodes in the network. Node a is the sending node, Nodes b and c are candidate forwarding nodes, and the set of candidate nodes is denoted by $S_{a\_u}=\{b,c\}$. $S_{b\_all}=\{a,x,y,z\}$ is the set of all the neighbor nodes of the candidate forwarding node b and $S_{c\_all}=\{a,k,l,m,n,p\}$ is the set of all neighbor nodes of the candidate forwarding node c. $S_{b\_u}=\{x,y\}$ is the set of upper-layer neighbor nodes of b. $S_{c\_u}=\{k,l,m,n\}$ is the set of upper-layer neighbor nodes of c.

(1) The linguistic values (i.e., fuzzy sets) are set for ND, CTV, and FP, respectively, as shown in Table 3.
(2) By using the triangular membership function and the trapezoidal membership function, membership functions of linguistic values of ND and CTV are fuzzified and expressed as shown in Equations (14)–(16):
  (14) $$f_{\min}(x)=\{\begin{array}{cc} 1, & x\leq a\\ \frac{b-x}{b-a}, & a\leq x\leq b\\ 0, & x\geq b \end{array}$$
  (15) $$f_{\mathrm{med}\;}(x)=\{\begin{array}{cc} 0, & x\leq e\\ \frac{x-e}{f-e}, & e\leq x\leq f\\ \frac{g-x}{g-f}, & f\leq x\leq g\\ 0, & x\geq g \end{array}$$
  (16) $$f_{\max}(x)=\{\begin{array}{cc} 0, & x\leq m\\ \frac{x-m}{n-m}, & m\leq x\leq n\\ 1, & x\geq n \end{array}$$
  where *a* = 0.33, *b* = 0.6, *e* = 0.33, *f* = 0.5, *g* = 0.67, *m* = 0.5, *n* = 0.67 for ND; *a* = 0.4, *b* = 0.6, *e* = 0.4, *f* = 0.6, *g* = 0.8, *m* = 0.6, *n* = 0.8 for CTV.

**Figure 7:**
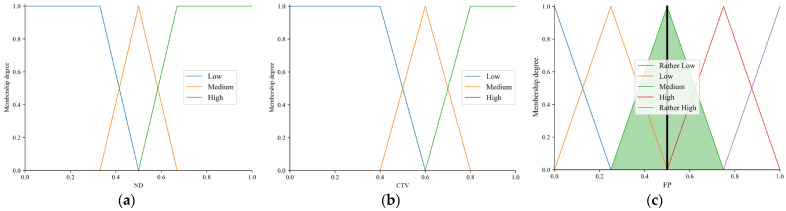
Membership functions. (**a**) Membership functions of ND. (**b**) Membership functions of CTV. (**c**) Membership functions of FP.

The membership functions of the ND and CTV are plotted, respectively, in Figure 7a,b.

**Figure 8:**
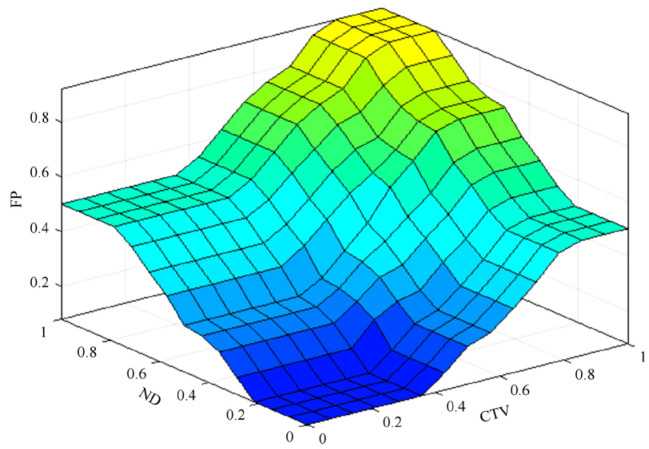
The relationship between input variables and output variable.

(3) Five linguistic values are set for the output variable FP: Rather Low, Low, Medium, High, and Rather High. The triangular membership function is used to fuzzify the FP, which is shown in Figure 7c. Figure 8 shows the relationship between the input variables ND and CTV and the output variables FP.

(4) Establish the rules of fuzzy control. The if–then rule is adopted in the FCS. The total fuzzy rules employed by the control engine are shown in Table 4. For example, if the ND is “Medium” and the CTV is “High”, the FP of the candidate forwarding node is “High”.
(5) FP is obtained in this step through defuzzification. The center of gravity method is used to find the center of the area enclosed by the curve of membership degree function and the *x*-axis. The value of the horizontal coordinate corresponding to the center is the output value FP. Assume that the domain $X_{fp\;}=\left\{x_{1},\;x_{2},\;\ldots ,\;x_{n}\right\}$ is discrete, and *f*(*x_i_*) is the membership degree of *x_i_*. Then, FP can be calculated via Equation (17).
  (17) $$FP=\frac{\sum_{i=1}^{n}\left(x_{i}f\left(x_{i}\right)\right)}{\sum_{i=1}^{n}f\left(x_{i}\right)}$$
(6) Select the best forwarding node. The sending node takes the candidate node with the largest FP in step (5) as the best forwarding node and sends the DATA packet to the best forwarding node.

**Figure 9:**
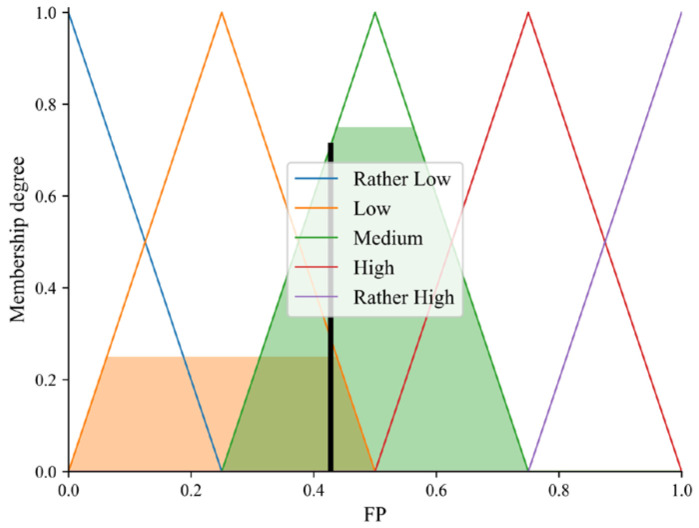
Membership degree functions and FP of the candidate node c.

**Figure 10:**
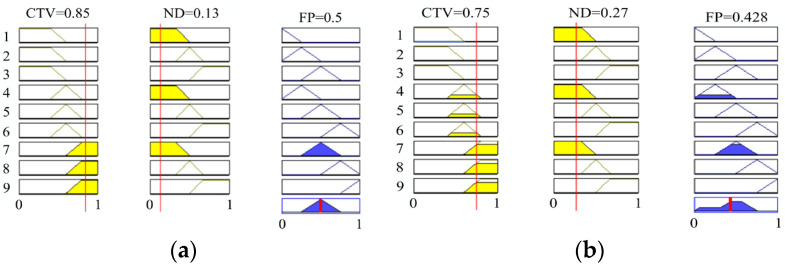
The rule triggering and defuzzification process. (**a**) The values of input variables are 0.85 and 0.13. (**b**) The values of input variables are 0.75 and 0.27.

Reviewing that the NDs of node b and node c are 0.13 and 0.27, respectively, assuming that the CTVs of node b and node c are 0.85 and 0.75, respectively, based on the above steps, it can be calculated that the FPs of node b and node c are 0.5 and 0.428, which are shown in Figure 7c and Figure 9, respectively. In addition, the rule triggering and defuzzification of node b and node c are simulated through the use of MATLAB R2022a, and illustrated in Figure 10a,b. Obviously, the FP of node b is greater than that of node c. Therefore, the sending node will select node b as the best forwarding node.

**Figure 11:**
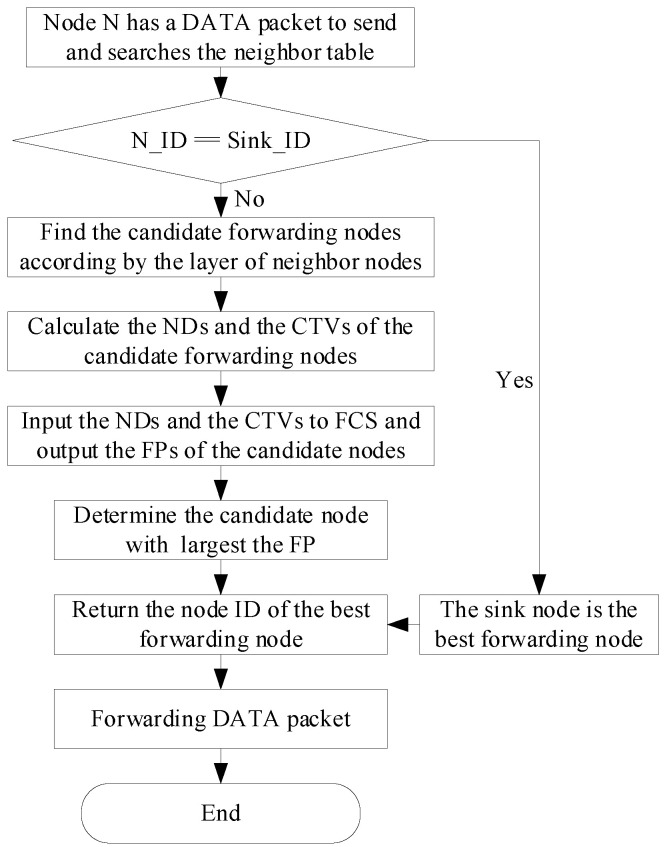
The forwarding flow of the TAFLRLR protocol.

After the best forwarding node is determined, the ID of the best forwarding node is filled in the field of the sender’s ID of the packet; then, the DATA packet is sent to the best forwarding node. The flow chart is shown in Figure 11, where the N_ID and Sink_ID represent the ID of the neighbor node and the ID of the sink node, respectively. From the flow chart, we can see the procedure includes the following five steps.

Step 1: When a sending node N has a DATA packet to send, it first searches its neighbor information table for the sink node. If it cannot find the sink node, Node N finds the nodes whose layers are 1 lower than its layer from its neighbor information table as candidate forwarding nodes.

Step 2: The sending node N calculates the CTV and ND for each candidate forwarding node.

Step 4: Determine the candidate forwarding node with the largest FP as the best forwarding node and return the ID of the best forwarding node.

Step 5: Node N updates the field of Rece ID in the packet with the ID of the best forwarding node and sends the DATA packet to the best forwarding node.

### 5.3. DATA Receiving

**Figure 12:**
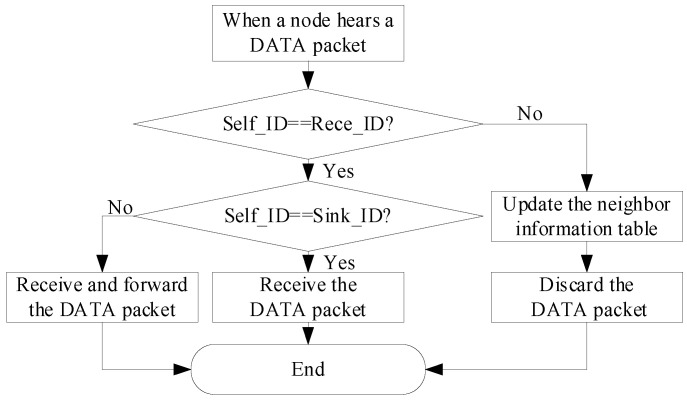
The receiving flow of the TAFLRLR protocol.

After Node N sends the DATA packet, the neighbor nodes within the transmission range of the sending node N can hear the DATA packet. The receiving flow of the DATA packet is shown in Figure 12, where Self ID represents the ID of the current receiving node and the Rece ID represents the ID of the destinated receiving node assigned by the head field of the packet. According to the Rece_ID, there are three cases of data receiving [17].

Case 1: If the node that hears the packet is a sink node, it updates the neighbor information table and receives the packet.

Case 2: If the ID of the receiving node is the same as the Rece_ID in the head of the packet, the receiving node updates the neighbor information table, receives the packet, and then finds the best next-hop forwarding node, updates the Rece_ID field of the packet with the ID of the best forwarding node and sends the packet to the best forwarding node.

Case 3: If the receiving node is neither the sink node nor the best forwarding node, it just updates the neighbor information table and discards the received packet.

## 6. Performance Evaluation

In this section, we evaluate the performance of the TAFLRLR protocol in terms of packet delivery rate (PDR) and total energy consumption (TEC) with the NS3 network simulation platform. The main simulation parameters are listed in Table 5.

### 6.1. Performance Metrics

Packet delivery rate: The ratio of the number of DATA packets delivered successfully to the sink node *N_sink_rece_* to the number of the DATA packets transmitted by the source nodes *N_source_*__send_ which is given by Equation (18) [17].

(18) $$PDR=\frac{\sum_{i=1}^{N_{simu\;}}\frac{N_{\sin k\_rece\;}}{N_{source}{}_{\_send\;}}}{N_{simu\;}}$$

where *N_simu_* is the number of simulation experiments.

Total energy consumption: The average of the total energy consumed by all nodes in the network in their operating states for *N_simu_* simulation experiments, which is given in Equation (19) [17].

(19) $$TEC=\frac{\sum_{i=1}^{N_{\mathrm{simu}\;}}\left(E_{\mathrm{send}\;}+E_{\mathrm{rece}\;}+E_{\mathrm{idle}\;}+E_{\mathrm{sleep}\;}\right)}{N_{\mathrm{simu}\;}}$$

where *E_send_* is the energy consumed by all nodes in the network in the sending state, *E_rece_* is the energy consumed by all nodes in the network in the receiving state, *E_idle_* is the energy consumed by all nodes in the network in the idle state, and *E_sleep_* is the energy consumed by all nodes in the network in the sleeping state.

### 6.2. Simulation Results Analysis

In this section, we analyze the performance of the TAFLRLR protocol under different simulation parameter settings. In the simulation experiments, the MNs employ mainly two attack methods: the HELLO packet flooding attack and the selective forwarding attack. Assuming that an MN can only use one attack mode. The MN with a selective forwarding attack directly discards the received packet. The MN with the first HELLO packet flooding attack forwards HELLO packets with a larger communication range than a normal node. By contrast, the MN with the second HELLO packet flooding attack forwards the received HELLO packets repeatedly.

#### 6.2.1. The Change of Trust Value of the Normal Node and MN

**Figure 13:**
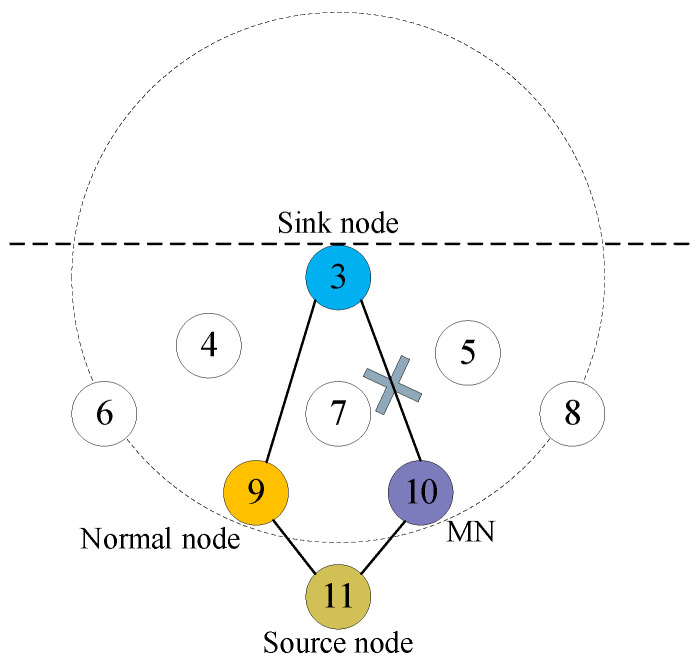
Experimental topology.

**Figure 14:**
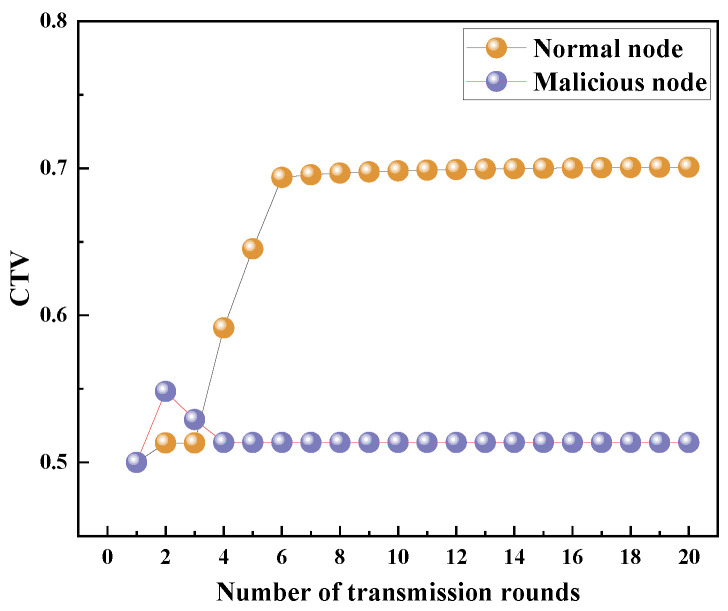
Trust values of a normal node and the MN.

The network topology in the experiment is shown in Figure 13. In Figure 13, nine nodes are deployed in the water. Most notably, node 10 is an MN with the selective forwarding attack mode. The trust values of the MN and a normal node, node 7, during 20 transmission rounds are recorded and they are shown in Figure 14.

From Figure 14, it is seen that as the number of transmission rounds increases, the trust value of the normal node gradually increases. When the number of transmission rounds exceeds six, the trust value of the normal node reaches 0.7 and then remains constant. In contrast, when the number of transmission rounds exceeds two, the trust value of the MN decreases to 0.52 and then remains constant. As mentioned earlier, the trust value is related to the residual energy of the node as well as the data packet forwarding behavior and the HELLO packet forwarding behavior. The trust value of the MN increases in the second transmission round because the MN forwards HELLO packets normally. However, after the second round, the trust value of the MN begins to decrease because the MN does not forward the received DATA packets. Since the nodes with low trust values have a small probability of being selected as the best next-hop forwarding node, the trust value of the MN tends to remain constant as the number of transmission rounds increases.

#### 6.2.2. PDR with FLTEM and without FLTEM of the Network with Stationary Topology

**Figure 15:**
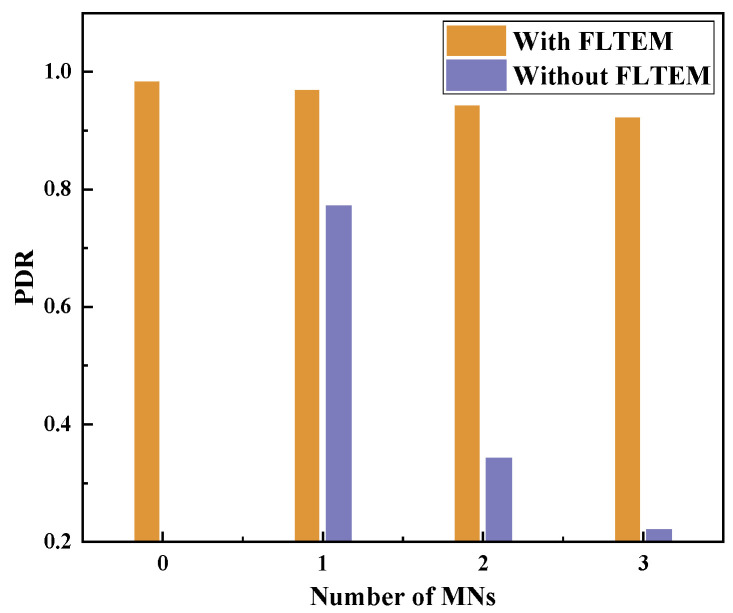
Effect of the number of MNs on PDR when the number of nodes is 9 and topology is fixed.

Based on the topology shown in Figure 13, we increase the number of MNs and compare the PDRs of the routing protocols with FLTEM and without FLTEM. Especially, the MNs can adopt an arbitrary attack method, as listed in Section 2. The experiment results are shown in Figure 15.

From Figure 15, it can be seen that when there are no MNs in the network, the PDR reaches 0.98. As the number of MNs increases, the PDR of the network, either with or without FLTEM, decreases. However, the PDR with FLTEM is generally greater than that without FLTEM. In addition, the PDR without FLTEM decreases sharply with the increasing number of MNs, while the PDR with FLTEM decreases slowly. The experimental results show that the FLTEM greatly improves the packet delivery rate and transmission reliability of the network with stationary topology.

#### 6.2.3. PDR with FLTEM and without FLTEM of the Network with Dynamic Topology

**Figure 16:**
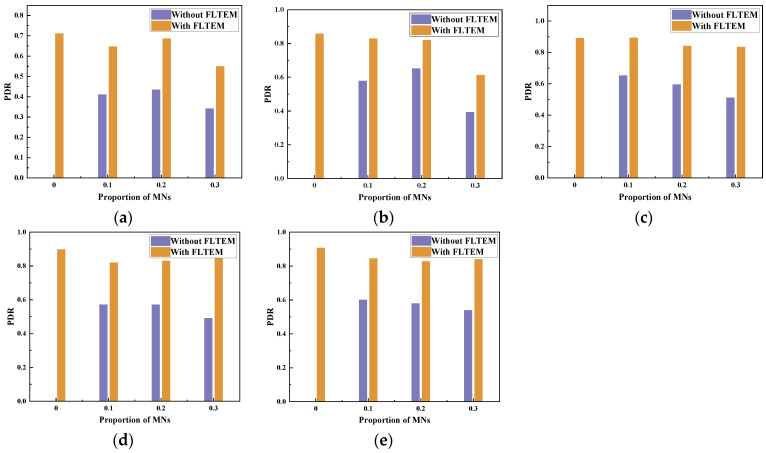
The effect of the number of MNs on PDR with or without FLTEM. (**a**) The total number of nodes is 40. (**b**) The total number of nodes is 100. (**c**) The total number of nodes is 160. (**d**) The total number of nodes is 220. (**e**) The total number of nodes is 300.

**Figure 17:**
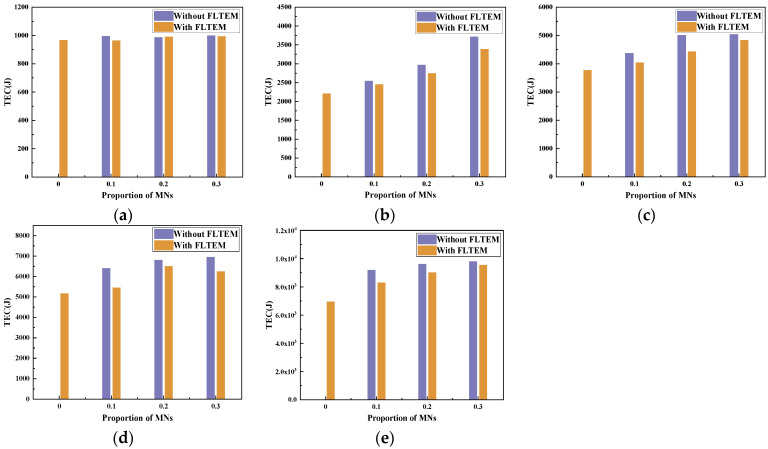
The effect of the number of MNs on TEC with or without FLTEM. (**a**) The total number of nodes is 40. (**b**) The total number of nodes is 100. (**c**) The total number of nodes is 160. (**d**) The total number of nodes is 220. (**e**) The total number of nodes is 300.

**Figure 18:**
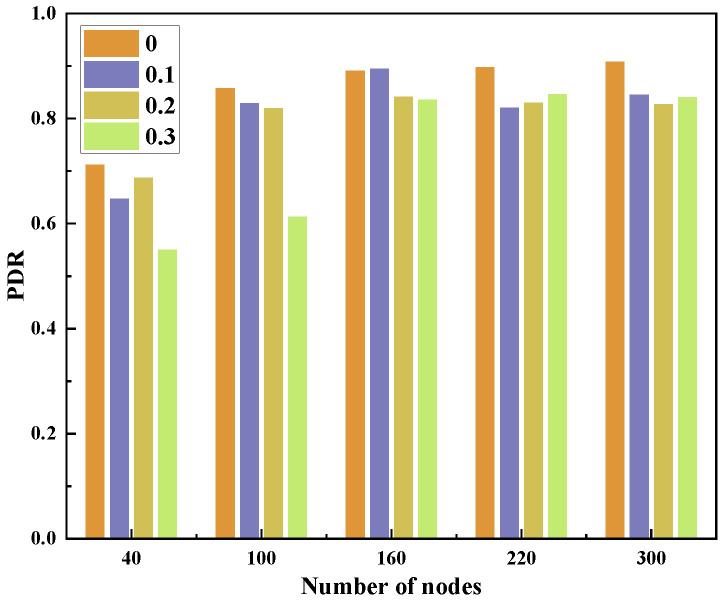
The effect of the number of nodes on PDR.

**Figure 19:**
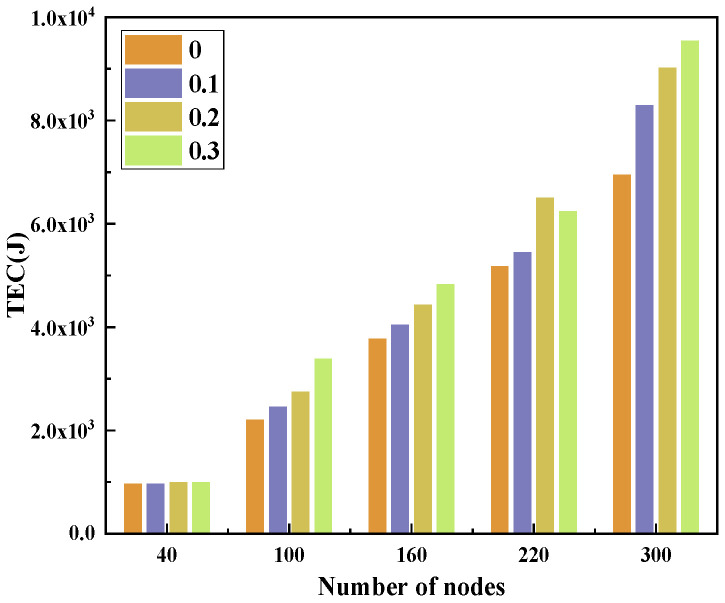
The effect of the number of nodes on TEC.

With the topology shown in Figure 1, we deploy 40–300 nodes in a 3D area of 500 m × 500 m × 500 m. We analyze the effect of FLTEM and the number of MNs on PDR and TEC. The proportion of MNs in the network is 0–0.3. Each MN has an arbitrary attack method listed in Section 2. The experimental results are shown in Figure 16, Figure 17, Figure 18 and Figure 19.

Figure 16a–e show the effect of the number of MNs on the PDR of the network with or without the FLTEM mechanism when the number of nodes is 40, 100, 160, 220, and 300, respectively. Specifically, the PDRs reach the maximum values when there is no MN in the network. When there are some MNs in the network, the PDRs decrease significantly. Meanwhile, the PDRs decrease with the increasing number of MNs. The more the number of MNs, the smaller the PDR is. It can be seen from Figure 16a–e that when there are MNs in the network, the PDRs with FLTEM are significantly improved and generally greater than those without FLTEM. The experimental results show that the FLTEM greatly improves the packet delivery rate and transmission reliability of the network with dynamic topology.

Figure 17a–e show the effect of the number of MNs on TEC with or without FLTEM when the number of nodes is 40, 100, 160, 220, and 300, respectively. From Figure 17a–e, it can be seen that TEC reaches the minimum value when there is no MN in the network. As the number of MNs increases, the TEC increases significantly. Moreover, it is also seen from Figure 17a–e that when there are some MNs in the network, the TEC without FLTEM is significantly greater than that with FLTEM. The experimental results show that the FLTEM mechanism can improve energy efficiency.

Figure 18 and Figure 19 show the effect of the number of nodes on PDR and TEC with FLTEM under the different proportions of MNs.

From Figure 18, we can see that no matter whether there are MNs in the network, the PDR increases with the number of nodes. Generally speaking, the PDR of the network without MNs is greater than the PDR of the network with MNs. The PDR increases as the number of MNs increases, and the PDR of the network with a large proportion of MNs is greater than that of the network with a small proportion of MNs.

From Figure 19, we can see that no matter whether there are MNs in the network, the TEC increases with the number of nodes. The TEC of the network with MNs is always greater than that of the network without MNs. Moreover, the TEC increases with the proportion of MNs in the network. This is because the number of HELLO packets flooded in the network increases with the number of nodes executing the HELLO packet flooding attack in Section 2, so the TEC of the network increases.

As shown in Figure 16, Figure 17, Figure 18 and Figure 19, the performance of the network in terms of PDR and energy efficiency significantly drops when there are some MNs in the network, which is unfavorable for UANs. Fortunately, the experimental results show that the performance of the network in terms of the PDR and energy efficiency is significantly improved after the FLTEM mechanism is introduced.

The above results show that both the trust mechanism FLTEM and the routing protocol TAFLRLR proposed in this paper can improve the PDR and reduce the TEC of the network.

## 7. Conclusions

In this paper, a trust mechanism FLTEM and reliable routing protocol TAFLRLR are proposed for UANs. Extensive simulation experiments are performed to evaluate the performance of the FLTEM and TAFLRLR protocols. We compare the performance of the network with and without FLTEM. The experimental results show that (1) The network without MN achieves the largest PDR and the lowest TEC. (2) The performance in terms of PDR and energy efficiency significantly drops when there are some MNs in the network. (3) The performance of the network with FLTEM is significantly improved compared with that without FLTEM. Therefore, the trust mechanism FLTEM and the routing protocol TAFLRLR proposed in this paper can improve the PDR and reduce the TEC of the network.

In future research, we will further optimize the FLTEM mechanism and the TAFLRLR protocol by using deep reinforcement learning, swarm intelligence algorithms, and clustering algorithms to further improve the PDR, energy efficiency, and reliability of the network.

## Figures and Tables

**Table 1:** The format of the HELLO packet.

Bits	8	16	8	16	2	…	16	8	…
Fields	Layer	S_ID	Layer	R_ID	Packet type 0:DATA 1: ACK 2: HELLO	…	Er	ND	…
	SN		RN						
Position	Head						Load		

**Table 2:** The structure of the neighbor information table.

ID	*L_n_*	*E_r_*	*N_n_c_*	*N_a_c_*	*N_n_d_*	*N_a_d_*	…
3	0	*E_r_*_3_	*N_n_c_*_3_	*N_a_c_*_3_	*N_n_d_*_3_	*N_a_d_*_3_	….
17	1	*E_r_*_17_	*N_n_c_*_17_	*N_a_c_*_17_	*N_n_d_*_17_	*N_a_d_*_17_	…
41	2	*E_r_*_41_	*N_n_c_*_41_	*N_a_c_*_41_	*N_n_d_*_41_	*N_a_d_*_41_	…
105	3	*E_r_*_105_	*N_n_c_*_105_	*N_a_c_*_105_	*N_n_d_*_105_	*N_a_d_*_105_	…
⋮	⋮	⋮	⋮	⋮	⋮	⋮	

**Table 3:** Fuzzy sets for ND, CTV, and FP.

Input/Output Variables	Linguistic Values				
ND	Low	Medium	High	-	-
CTV	Low	Medium	High	-	-
FP	Rather Low	Low	Medium	High	Rather High

**Table 4:** Fuzzy rule table in TAFLRLR protocol.

Rule	ND	CTV	FP
1	Low	Low	Rather Low
2	Low	Medium	Low
3	Low	High	Medium
4	Medium	Low	Low
5	Medium	Medium	Medium
6	Medium	High	High
7	High	Low	Medium
8	High	Medium	High
9	High	High	Rather high

**Table 5:** Simulation parameter setting.

Simulation Parameter	Value	Unit
Simulation scene range	500 × 500 × 500	m
Simulation time	800	s
Proportion of MNs	10–30%	
DATA packet size	154	Bytes
The moving speed of the nodes	1–3	m/s
Transmitting power	2.0	w
Receiving power	0.1	w
Idle power	0.01	w
Topology	Fixed/Random	
Initial energy	1000	J
Number of experiments	30	

## Data Availability

Data are contained within the article.
